# Snoring Prevented by Excision of the Uvula

**Published:** 1852-11

**Authors:** 


*Snoring Prevented by Excision of the Uvula.* By THE EDITOR.

CASE. A. D——, about five years of age, had for two or three years suffered from considerable enlargement of the tonsils, which impeded respiration so much during sleep, as to cause him to snore very loudly, and to seem to be on the point of suffocation. About a year ago, I excised both tonsils, after which the respiration was very much improved, and the snoring nearly ceased. In March last, his respiration during sleep had become as bad as ever, and his parents apprehending that he might actually suffocate, again requested medical aid. Upon examination, I found that the tonsils had again become somewhat enlarged; that the uvula hung flabbily between them and rested upon the base of the tongue, and that this state of things taken in connection with the natural, yet extraordinary smallness of the bucco-pharyngeal aperture, was sufficient to account for the impediment in respiration. It should be remarked, however, that, although the uvula appeared flabby, it was not paralyzed, for it would sometimes retract spontaneously, and always do so when touched with an instrument.

As the tonsils projected but slightly beyond their proper limits, and their further excision was very difficult, if not hazardous, in consequence of the smallness of the mouth and extreme narrowness of the throat, I resolved to try the effect of simply clipping off the uvula. The child has not snored since, and has from that time slept without any impediment in his respiration.

Would it not be advisable to resort to this simple operation for the relief of snoring in adults? It is certainly worthy of trial, and might add very much to the comfort of those who are annoyed by a snoring bed-fellow.—*Southern Med. and Surg. Journal.*

